# Successful hemodialysis catheter placement into a stenosed femoral vein using balloon dilation for central venous access

**DOI:** 10.1016/j.radcr.2025.04.119

**Published:** 2025-05-27

**Authors:** Koichi Saiki, Takuro Fujita, Yoshihiro Toyama, Tomoko Yokoyama, Masahito Yamanaka

**Affiliations:** a Department of Nephrology, Takamatsu Red Cross Hospital, Takamatsu, Japan; b Department of Radiology, Takamatsu Red Cross Hospital, Takamatsu, Japan; c Department of Urology, Takamatsu Red Cross Hospital, Takamatsu, Japan

**Keywords:** Hemodialysis, Central venous catheter, External iliac vein, Vascular access, Central venous stenosis

## Abstract

A 67-year-old woman with end-stage renal failure due to immunoglobulin A nephropathy had undergone hemodialysis for 20 years. The patient was dependent on catheterized dialysis and underwent 13 central venous catheter changes. A GlidePath catheter (Bard Medical, Georgia, USA) was placed in the right femoral vein and removed because of infection. There was no alternative but to implant the central venous catheter at the same site. Computed tomography revealed stenosis of the right external iliac vein. Therefore, balloon dilation with a Mustang balloon (5.0 mm) (Boston Scientific, Marlborough, Massachusetts, USA) was performed before central venous catheter implantation. The number of patients undergoing dialysis who require vascular access catheters is expected to increase because of the aging population and extended duration of dialysis treatments. Balloon dilation prior to catheter placement is effective in patients with vascular stenosis.

## Introduction

Vascular access must be secured for hemodialysis (HD). An arteriovenous fistula (AVF) is typically created on the forearm. Other options include placement of a central venous catheter (CVC) or arteriovenous graft (AVG). If an AVF cannot be created, CVC are often implanted. However, repeated removal and reimplantation due to infection or other complications can lead to vessel stenosis, making subsequent implantation challenging. Catheter placement in stenotic vessels should be avoided; however, prolonged dialysis may require catheter placement in such vessels. If severe stenosis prevents catheter placement, balloon catheter dilation or stenting may be performed [1]. Although numerous studies are available on central venous stenosis (CVS) of the superior vena cava, including CVS incidence and recurrence rates, few reports have addressed stenosis of the inferior vena cava (IVC) and external iliac vein caused by CVC implantation into the femoral vein and balloon dilatation [2].

In addition, previous studies have mainly employed balloon dilation under the assumption of stent placement. However, this approach carries the risk of vascular injury due to large-diameter balloon dilation; therefore, small-diameter balloon dilation may reduce the risk of vascular injury during catheter placement. In this case, frequent CVC replacement led to stenosis of the external iliac vein; therefore, balloon dilation was performed before CVC placement, allowing the procedure to be completed without complications.

## Case report

A 67-year-old woman had been on hemodialysis for 20 years prior because of end-stage renal failure secondary to immunoglobulin A nephropathy. After initiating hemodialysis, a left-forearm AVF was created and used.

The patient had an AVG in the right forearm due to AVF failure, which was removed because of infection. Subsequently, the patient was dialyzed using a CVC. Frequent infections and dysfunction occurred after CVC implantation, and the CVC was replaced 13 times.

During dialysis, the patient developed shivering, fever, and pus drainage at the site of the catheter insertion. Computed tomography (CT) revealed fat stranding around the CVC outlet. Following thorough examination, a diagnosis of CVC infection was made. The laboratory findings at admission are displayed in Table 1.

**Table 1:** Laboratory examination on admission (after dialysis).

CRP	22.7	mg/dL
BUN	11.2	mg/dL
Creatinine	1.45	mg/dL
White blood cells	10570	/µL
Neutrophilis	89.9	%
Platelets	24.0	10^4^/µL
PT-INR	0.96	
APTT	32.9	seconds
Blood culture	*Staphylococcus aureus*	(1+)

Coagulation system was tested the day after admission.

APTT, Activated partial thromboplastin time; BUN, Blood urea nitrogen; CRP, C-reactive protein; PT-INR, Prothrombin time-international normalized ratio.

The catheter was removed, and antibiotic therapy was initiated for CVC infection of the right femoral vein. Contrast-enhanced CT was performed to evaluate vascular access after CVC extraction. Severe stenosis of the bilateral jugular and subclavian veins, diffuse IVC stenosis, and new thrombus formation in the intrahepatic IVC were observed.

**Fig. 1:**
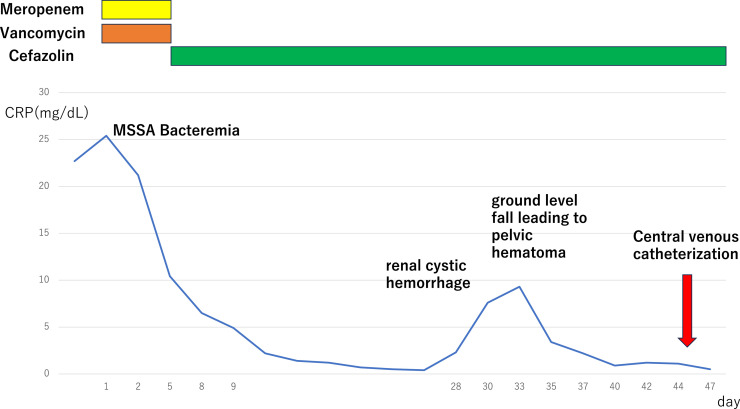
Clinical course after hospitalization. A temporary central venous catheter (CVC) is inserted into the right femoral vein for hemodialysis until a new tunneled CVC was placed.

After initiating warfarin potassium (Warfarin tablets, Eisai, Tokyo Japan) at 1.0 mg/day, the prothrombin time-international normalized ratio (PT-INR) immediately increased to 5.0. After reducing the warfarin potassium dose to 0.5 mg/day, the PT-INR stabilized at 4.0, remained elevated, and was difficult to control. After the PT-INR stabilized, an idiopathic right renal cyst and pelvic hemorrhage occurred due to a ground-level fall. The patient was conservatively treated with blood transfusion. A schematic summary of the clinical course is provided in Fig. 1.

**Fig. 2:**
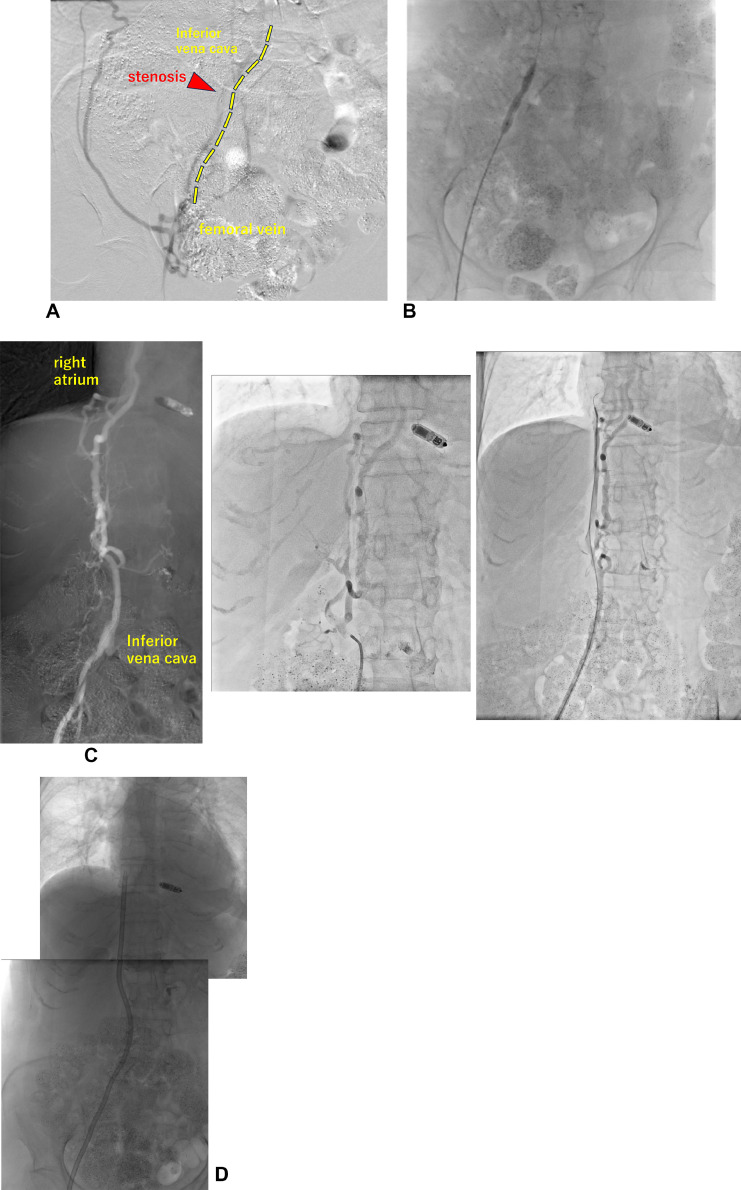
(A, B) Puncture of the right femoral vein and contrast display stenosis in the external iliac vein; balloon dilation with Mustang 5.0 mm is performed. (C) Contrast imaging from the inferior vena cava to the right atrium. Development of branch veins and meandering of vessels are observed. No thrombus is noted in the intrahepatic inferior vena cava. (D) After placement of 42-cm GlidePath catheter. The catheter tip can be observed in the right atrium.

After the patient's condition improved, another CVC was planned to be inserted through the right femoral vein. However, the right external iliac vein became stenotic due to frequent catheter placement. Thrombus formation was observed (Fig. 2).

Due to the difficulty in puncturing the left femoral vein, an introducer IIH 5Fr (TERUMO, Tokyo, Japan) was placed from the right femoral vein, and contrast imaging was performed. The external iliac vein narrowed as it extended to the IVC and collateral branches developed. A 35-inch Radifocus guidewire M (TERUMO, Tokyo, Japan) was advanced first, followed by the placement of a Mustang 5.0-mm balloon at the stenosis of the external iliac vein. Satisfactory dilatation was observed. The wire was advanced into the right atrium in conjunction with 4Fr TEMPO4 (Cordis, California, USA).

The patient was transported to CT for confirmation, which confirmed that the pathway passed through the IVC. The entire IVC was dilated with a Mustang 5.0-mm balloon, followed by a 7.0-mm balloon. No apparent stenosis was observed. After creating a pocket at the puncture site and performing dilation with a 10Fr sheath, a 14.5Fr GlidePath catheter was placed near the right atrium. The blood draw was successful, and dialysis was performed without difficulty during the first 6 months after catheter intervention.

## Discussion

In this patient, a Bioflex catheter (Hayashidera, Ishikawa, Japan) was initially placed at the level of the renal vein from the confluence of the IVC. However, owing to recurrent catheter dysfunction caused by vascular stenosis and thrombus formation, a 42-cm GlidePath catheter was inserted into the right atrium.

Thrombus formation in the intrahepatic IVC may have been introduced by a Bioflex catheter positioned with the tip at the level of the renal vein confluence. Because repeated CVC placement caused stenosis of the central vein, balloon dilation was performed before the placement of a new CVC.

In previous reports, balloon dilation was often employed before stent placement, with balloon diameters reaching up to 12 mm in preparation for the final stent placement, ranging from 12 to 18 mm [3,4]. In order to intervene with the expectation of improving venous stasis, the largest-diameter balloon was used [3,4].

In this case, only a vessel diameter large enough to accommodate the catheter was required.

No significant problems with venous return were observed in the development of collateral blood vessels. Considering the risk of complications, including central venous injury, we limited the balloon size during dilation to the size of the catheter. The GlidePath catheter used in this case was 14.5Fr and had an outer diameter of 4.8 mm.

GlidePath catheters lack sheaths of sufficient length and vascular stenosis in the IVC may lead to vascular injury during placement. Balloon dilation to a diameter of approximately 7.0 mm would limit the risk of vascular injury . Not used in this case, the Peel-Away Sheath Introducer (Cook Medical, Kanosha, USA) was 16Fr, 30 cm, and the use of the sheath would limit the risk of vascular injury.

Fibrin sheaths may develop around the CVC, with an incidence of formation ranging from 13% to 57% [5]. The mechanism of fibrin sheath formation is as follows: first, a protein layer of approximately 100 nm composed of fibrinogen, albumin, gamma globulin, lipoproteins, and coagulation factors forms at the venous insertion site on the catheter surface and extends along the catheter. Some studies have indicated that the fibrin sheath can cover the entire catheter in approximately 1 week [5]. Albumin induces the conversion of fibrinogen to fibrin, which eventually transforms into fibrous connective tissue [5].

Second, smooth muscle cells of the venous wall migrate toward the intimal layer, resulting in collagen deposition. If the fibrin sheath forms a valve-like structure at the catheter tip, it may allow blood infusion while obstructing blood aspiration, potentially resulting in catheter malfunction [[5], [6], [7]].

Hemodialysis with CVCs can result in complications, including infection, blood clots, and CVS. Formation of a fibrin sheath on the CVC increases the risk of catheter infection and persistent bacteremia. In a previous study, the presence of anticoagulants, antiplatelet medications, and prothrombin time did not influence the formation of fibrin sheaths on catheter [8].

In a study of tunneled CVC using the right and left internal jugular veins, the incidence of CVS progressively increased with a longer duration of CVC placement, with rates of 10%, 12%, 24%, and 28% in patients with duration under 3, 3-6, 6-9, and 9 months or longer, respectively [2]. The occurrence of CVS is higher in patients who undergo long-term indwelling catheter insertion [2].

A meta-analysis of 45 patients with iliofemoral vein obstruction who underwent balloon dilation and stenting reported rare complications. Major bleeding during central venous catheter insertion has been reported in 0.3%-1.1% of cases, including wound hematomas, common femoral artery puncture during sheath insertion, and access site bleeding requiring blood transfusion. Other complications included pulmonary embolism (0.2%-0.9%) and perioperative mortality 0.1%-0.7% [9]. The primary and secondary patency of catheters was higher in patients with nonthrombotic occlusions than in those with acute or chronic thrombotic occlusions [9].

Trans-lumbar and trans-hepatic catheterization were also considered. Translumbar catheters are thought to have higher infection rates because their exit sites are located on the back, and do not last more than 2 months without replacement owing to associated complications [10].

Transhepatic catheters are similarly open for approximately 87.7 days and are prone to malfunction due to abnormal positioning of the catheter caused by breathing. Hepatic hemorrhage occurs in 29% of catheter insertion cases [10]. Both accesses are recommended for short-term use while waiting for the AVF and graft to mature [10,11].

## Conclusion

With an aging population and prolonged dialysis duration, the number of patients with CVCs in the femoral vein is expected to increase.

In cases of venous stenosis resulting from repeated CVC placement, balloon dilatation combined with CVC reinsertion may facilitate this procedure.

## Ethical approval

All procedures involving human participants performed in this study were in accordance with the ethical standards of the institutional and national research committees, the Declaration of Helsinki of 1964, and its subsequent amendments or comparable ethical standards.

## Patient consent

Informed consent was obtained from individual participants in the study.
